# The causal relationship between allergic diseases and heart failure: Evidence from Mendelian randomization study

**DOI:** 10.1371/journal.pone.0271985

**Published:** 2022-07-29

**Authors:** Yan-Ge Guo, Yan Zhang, Wei-Li Liu

**Affiliations:** Fuwai Central China Cardiovascular Hospital, Heart Center of Henan Provincial People’s Hospital, Central China Fuwai Hospital of Zhengzhou University, Zhengzhou, Henan, China; Massachusetts General Hospital/Harvard Medical School, UNITED STATES

## Abstract

**Background:**

Emerging evidence shows allergic diseases, such as atopic dermatitis and asthma, are risk factors of heart failure. However, the causal relationship between allergic diseases and heart failure is not clear.

**Methods:**

We performed a two-sample Mendelian randomization analysis between allergic diseases and heart failure using summary statistics of genome-wide association studies from large GWAS consortia, with total sample size of 1.2 million. Independent instrumental variables for asthma and atopic dermatitis (P<1×10^−5^) were used as the exposure. We applied five models for the Mendelian randomization analysis. Finally, we performed the sensitivity analyses to assess the robustness of the results.

**Results:**

We have identified 55 independent single nucleotide polymorphisms (SNPs) for asthma 54 independent SNPs for atopic dermatitis as our instrumental variables. The inverse variance-weighted (IVW) analysis showed asthma was significantly associated with increased risk of heart failure (OR_IVW_ = 1.04, 95% CI, 1.01–1.07, P = 0.03). The Mendelian randomization analysis using the other four models also showed consistent results with the IVW analysis. Similarly, atopic dermatitis was also significantly associated with an increased risk of heart failure (OR_IVW_ = 1.03, 95% CI, 1.01–1.06, P = 0.01), consistent with the other four models. The sensitivity analysis showed no evidence of horizontal pleiotropy or results were driven by single SNPs.

**Conclusion:**

Our study identified asthma and atopic dermatitis as a causal risk factor for heart failure and suggest inflammatory pathogenesis as a key factor contributing to the underlying mechanism. These findings emphasize the importance of asthma and allergy control in the prevention and management of heart failure.

## Introduction

Heart failure is a complex and deadly disease affecting at least 26 million people globally, which exerts a large public health burden [1]. Heart failure is defined as a chronic syndrome with pulmonary or systemic congestion due to structural and/or functional cardiac abnormalities [2]. Although advances in the prevention of heart failure, the mechanism of comorbidities and heart failure remain unclear. Recent observational studies have found allergic diseases, such as atopic dermatitis and asthma, are associated with heart failure [3–6]. However, it is unknown whether there is a causal relationship between allergic diseases and heart failure.

Mendelian randomization (MR) is a method using genetic variants as the instrumental variable (IV) to investigate the causal relationship between exposure and outcome. The advantage of MR over conventional observational studies is that the MR method can minimize the likelihood of confounding and remove reverse causality [7, 8]. One commonly used MR method is called two-sample MR [9]. This method utilizes genetic data (e.g., genome-wide association study [GWAS] summary statistics) from two different study samples to estimate the causal effect of exposure (i.e., risk factors) and outcome.

Both allergic diseases and heart failure are complex diseases with relatively high genetic influence [10–14]. For example, heritability can range from 71% to 84% for atopic dermatitis [15], 35% to 95% for asthma [15], and 26% to 34% for heart failure [16, 17]. Thus, we hypothesized that allergic diseases, such as atopic dermatitis and asthma, have a genetic causal effect on heart failure. In the current study, by using recently published large-scale GWAS summary statistics data and a two-sample MR approach, we aim to explore the causal relationship between atopic dermatitis, asthma, and heart failure.

## Methods

### Study design, assumptions, and GWAS data

**Fig 1:**
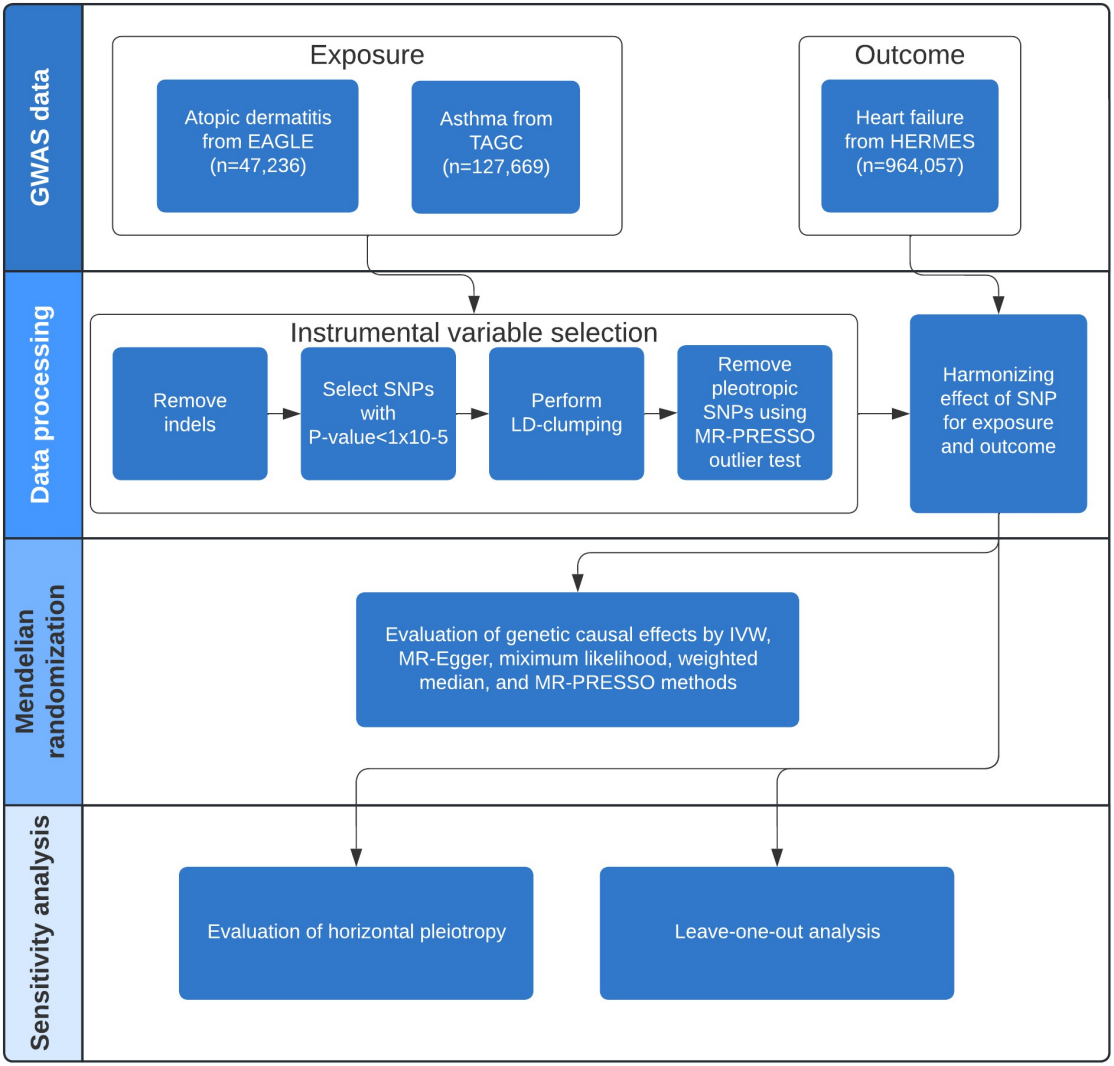
Study design, data preparation and analytical flow.

**Fig 2:**
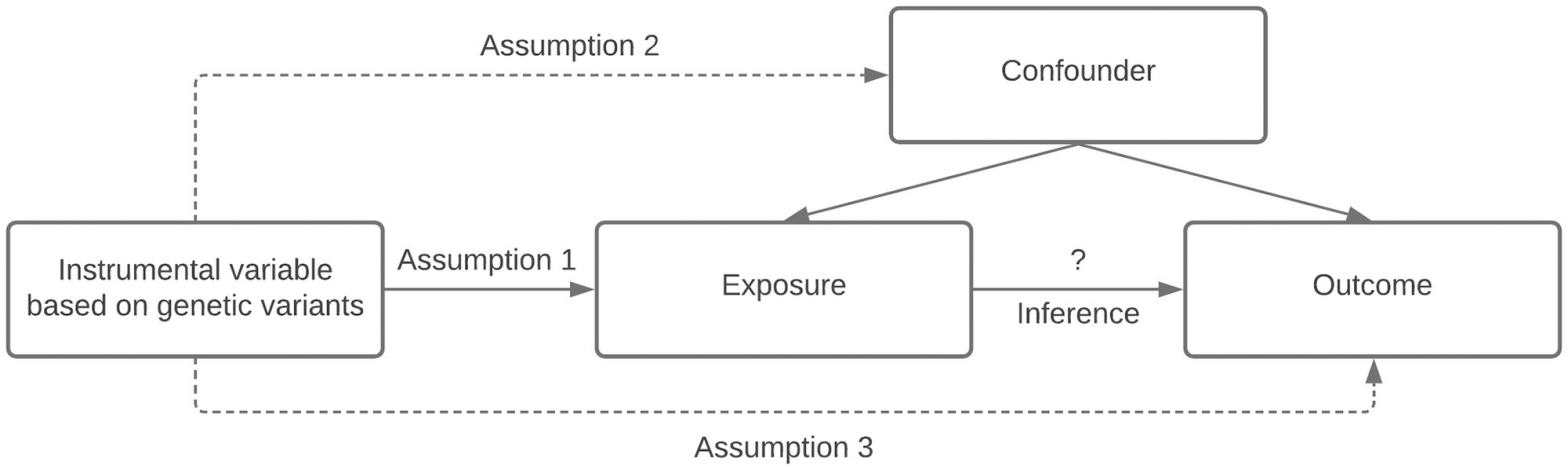
Three key assumptions in Mendelian randomization study.

We performed the current study using a two-sample Mendelian randomization analysis, where instrumental variable from the exposure and outcome associations were extracted from two independent non-overlapping sets of participants. The detailed study design of our analysis can be found in Fig 1. In addition, Mendelian randomization depends on three key assumptions to maintain validity of the analysis: 1) relevance assumption, i.e., instrumental variable based on genetic variants are associated with the exposure, 2) independence assumption, i.e., instrumental variable based on genetic variants are not associated with confounders, and 3) exclusion restriction, i.e., instrumental variable based on genetic variants influence outcome only through the exposure (Fig 2). The details of these assumptions can be found in the recent review studies [7, 8]. We have used the GWAS summary statistics from three data sources, asthma from the Trans-National Asthma Genetic Consortium (TAGC) [18], atopic dermatitis from the EArly Genetics and Lifecourse Epidemiology (EAGLE) Eczema Consortium [14], and heart failure from the Heart Failure Molecular Epidemiology for Therapeutic Targets (HERMES) Consortium [16]. To minimize genetic heterogeneity due to population stratification, we restricted the study samples to European only population. The asthma GWAS data contains 19,954 cases and 107,715 controls. The atopic dermatitis GWAS data contains 18,900 cases and 84,166 controls. The heart failure GWAS data contains 47,309 cases and 930,014 controls. All data was based on hg19 genome build and imputed using multiple reference panels. The detailed information of the GWAS data can be found in S1 Table.

### Exposure

The exposures of interest are asthma and atopic dermatitis. We have retrieved the GWAS summary statistics for asthma from TAGC consortium [18] and atopic dermatitis from EAGLE consortium [14]. Asthma cases were defined based on doctor’s diagnosis and/or standardized questionnaires, controls were participants without asthma [18]. Atopic dermatitis cases were defined based on doctor’s diagnosis (skin examination) or standardized questionnaires, and controls were participants without atopic dermatitis [14]. For instrumental variable selection process, first of all, we restricted our analysis to autosomal and biallelic variants. Second, to potentially increase the power and number of SNPs, we have used P-value<1×10^−5^ as the significance level to include SNPs into the instrumental variable. Then we have performed clumping analysis using default settings of “clump_data” function (—window-size 10,000 KB and—r2 0.001) and European subjects from the 1000 Genomes Project Phase 3 version 5 as the reference panel to select linkage-disequilibrium independent SNPs [19]. Finally, we checked and removed any SNPs that are palindromic, i.e., A/T or G/C alleles. The palindromic SNPs can introduce ambiguity of the effect allele between the exposure and outcome GWASs. Finally, we used Mendelian Randomization Pleiotropy RESidual Sum and Outlier (MR-PRESSO) global and outlier analyses to identify and remove SNPs with horizontal pleiotropic effects [20].

### Outcome

The outcome of interest is heart failure. We have retrieved the currently largest GWAS for heart failure from HERMES consortium [16]. Heart failure cases was defined based on doctor’s diagnosis, and controls were participants without heart failure. The heart failure GWAS data includes participants of European ancestry from 26 cohorts (with a total of 29 distinct datasets) with either a case-control or population-based study design were included in the meta-analysis, as part of the HERMES Consortium. From this GWAS summary statistics data, we extracted the variant information, including effect allele, non-effect allele, effect allele frequency, genomic coordinates, beta coefficients, standard error, and P-value.

### Mendelian randomization analysis

First of all, we harmonized the effect of SNP for both asthma or atopic dermatitis and heart failure before the Mendelian randomization analysis. Second, we evaluated a causal relationship between asthma and heart failure or atopic dermatitis and heart failure. To satisfy the Mendelian randomization assumption and comprehensively evaluate the causal effect between exposures and outcome, we applied a five Mendelian randomization approaches, inverse variance-weighted (IVW) method [21], MR–Egger regression method [22], maximum likelihood method [23], a weighted median method [24], and MR-PRESSO method [20].

### Sensitivity analysis

To evaluate the robustness of our Mendelian randomization results, we have performed two types of sensitivity analysis, pleiotropy test and leave-one-out analysis. Horizontal pleiotropy is defined as a gene that affects multiple traits or diseases, which is common in genetic studies of complex traits or diseases. The Mendelian randomization can be overestimated due to overall unbalanced horizontal pleiotropy. Thus, we conducted pleiotropy test after MR analysis to ensure that the results were free of horizontal pleiotropy. The influence of pleiotropic SNPs on MR analyses was evaluated by the MR-Egger intercept [22]. In addition, to investigate if a single SNP is driving the Mendelian randomization association, we performed Mendelian randomization again with leaving out each SNP in turn.

## Results

The current study included 127,669 subjects from asthma GWAS, 103,066 subjects from atopic dermatitis GWAS, and 977,323 subjects from heart failure GWAS. All subjects were European ancestry. There was a total of 2,001,280 SNPs in asthma GWAS data, 11,296,420 SNPs in atopic dermatitis GWAS data, and 8,281,262 in heart failure GWAS data (S1 Table).

Based on instrumental variable criteria, we have identified 55 independent SNPs for asthma 54 independent SNPs for atopic dermatitis as our instrumental variables and extracted the SNP information, including effect allele, non-effect allele, effect allele frequency, genomic coordinates, beta coefficients, standard error, and P-value from the asthma and atopic dermatitis GWASs. Details on characteristics of the instrumental variables can be found in S2 and S3 Tables.

**Fig 3:**
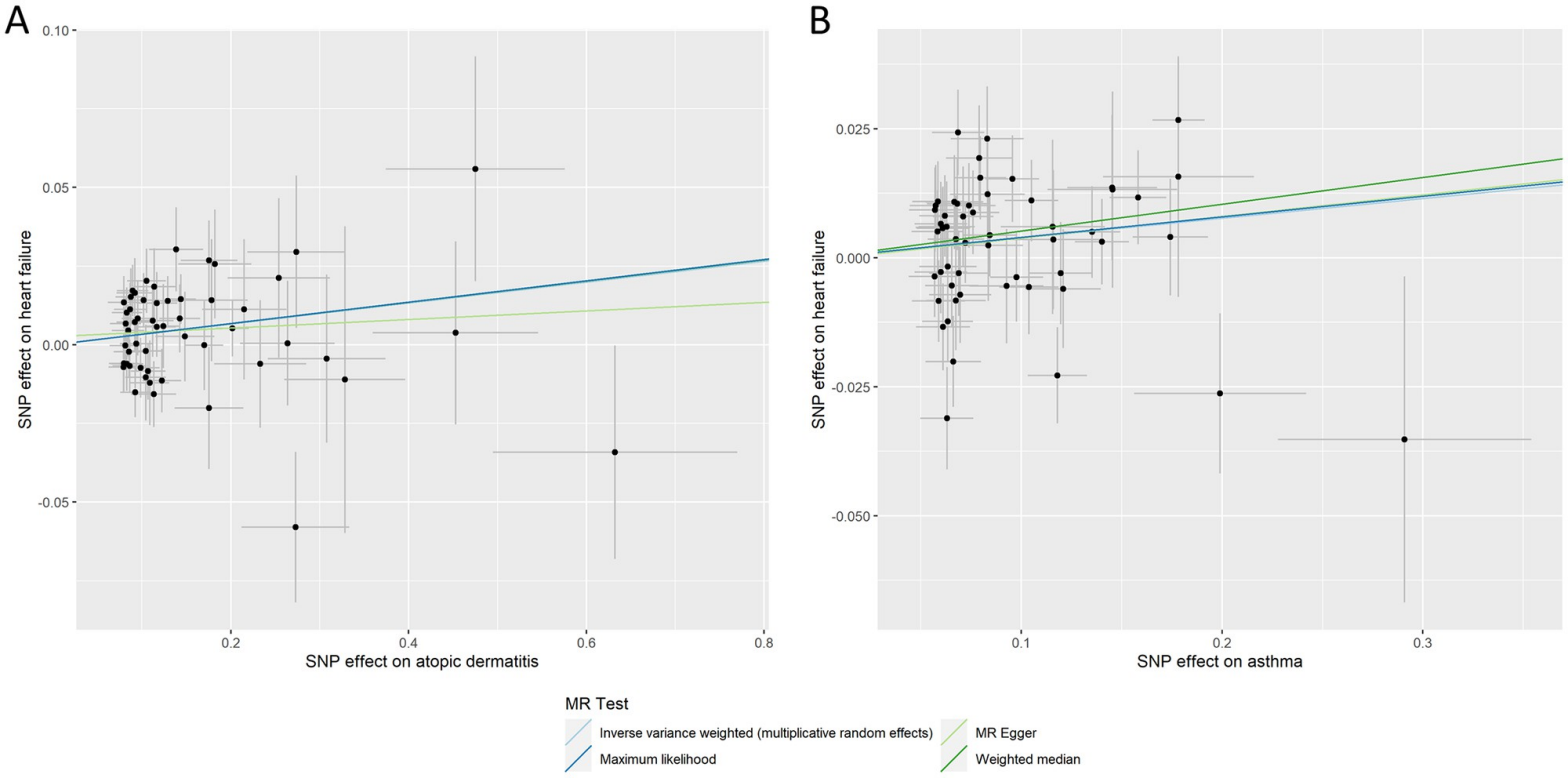
Instrumental variable SNP effect estimates between atopic dermatitis or asthma and heart failure.

The IVW results showed asthma was significantly associated with increased risk of heart failure (OR_IVW_ = 1.04, 95% CI, 1.01–1.07, P = 0.03) (Table 1 and Fig 3). The Mendelian randomization analysis based on the other four models also showed consistent results with the IVW model, with some of the methods remained significant, such as maximum likelihood method (OR_ML_ = 1.04, 95% CI, 1.01–1.06, P = 0.0005) and MR-PRESSO (OR_MR-PRESSO_ = 1.03, 95% CI, 1.01–1.06, P = 0.01) (Table 1). However, MR-Egger and weighted median methods did not show significant result. Similarly, atopic dermatitis was also significantly associated with increased risk of heart failure (OR_IVW_ = 1.03, 95% CI, 1.01–1.06, P = 0.01) (Table 1 and Fig 3), consistent with the 4 methods from secondary Mendelian randomization analysis, maximum likelihood method (OR_ML_ = 1.04, 95% CI, 1.01–1.07, P = 0.0004), weighted median method (OR_WE_ = 1.05, 95% CI, 1.01–1.10, P = 0.02), and MR-PRESSO (OR_MR-PRESSO_ = 1.04, 95% CI, 1.01–1.07, P = 0.03) (Table 1). In addition, we did not identify evidence of horizontal pleiotropy by MR–Egger regression (asthma-heart failure MR–Egger intercept P = 0.93; atopic dermatitis-heart failure MR–Egger intercept P = 0.52). The leave-one-out analysis did not identify any single SNP drive the overall causal association (S4 and S5 Tables).

**Table 1:** Mendelian randomization analysis of atopic dermatitis or asthma with heart failure using IVW, MR Egger, Weighted median and Weighted mode methods.

Exposure	Outcome	Method	Odds ratio (95% CI)	P-value
Asthma	Heart failure	IVW	1.04 (1.01–1.07)	0.03
Asthma	Heart failure	MR-Egger	1.04 (0.95–1.14)	0.38
Asthma	Heart failure	Maximum likelihood	1.04 (1.01–1.07)	0.004
Asthma	Heart failure	Weighted median	1.05 (1.01–1.10)	0.02
Asthma	Heart failure	MR-PRESSO	1.04 (1.01–1.07)	0.03
Atopic dermatitis	Heart failure	IVW	1.03 (1.01–1.06)	0.01
Atopic dermatitis	Heart failure	MR-Egger	1.01 (0.95–1.08)	0.68
Atopic dermatitis	Heart failure	Maximum likelihood	1.04 (1.01–1.06)	0.005
Atopic dermatitis	Heart failure	Weighted median	1.03 (1.00–1.07)	0.07
Atopic dermatitis	Heart failure	MR-PRESSO	1.03 (1.01–1.06)	0.01

Abbreviations: IVW, inverse variance weighted; MR, Mendelian randomization; MR-PRESSO, Mendelian Randomization Pleiotropy RESidual Sum and Outlier.

## Discussion

Heart failure remains a leading cause of cardiovascular mortality, with its high prevalence and public health burden globally [1]. Given the underlying inflammatory characteristics of heart failure with its comorbidities [25, 26], growing evidences suggest that allergic disease is a risk factor for heart failure [3–6], but the evidence on the causal relationship of asthma and atopic dermatitis on heart failure risk is limited. In the present MR study, we used genetic instrumental variable derived from the currently largest and independent asthma and atopic dermatitis GWASs to investigate the causal relationship of allergic diseases with heart failure. We found causal associations of both asthma and atopic dermatitis with heart failure.

There are several mechanisms to explain the causal association of asthma with heart failure. Heart failure is a state of chronic inflammation, characterized by heightened levels of circulating and myocardial pro-inflammatory cytokines that promote pathological left and right ventricular remodeling [27]. The activated inflammatory state in asthma patients may contribute to the pathogenesis of heart failure. First of all, chronic allergic lung inflammation causes remodeling of extra-bronchial lung vasculature [28], which is associated with higher risk for the development of pulmonary hypertension [29]. Elevated pulmonary artery pressure and pulmonary vascular resistance (PVR) will increase right ventricular afterload and eventually lead to right-sided heart failure [30]. Secondly, patients with asthma have increased levels of plasma cytokines such as IL-4, IL-5, and IL-13 [31] and chemokines such as monocyte chemoattractant protein-1 (MCP-1) [32]. For example, IL-13 can promote wound healing in myocardium after myocardial infarction, decrease LV dilation and increase LV function, and prevent heart failure [33]. Also, eosinophil-derived IL-4 can drive the progression of myocarditis to inflammatory dilated cardiomyopathy, which is a major cause of heart failure in children and young adults [34]. A previous study also indicated the direct roles of MCP-1 in the development of heart failure [32]. In transgenic mice with myocardial overexpression of MCP-1, leukocyte infiltration into interstitium between cardiomyocytes was increased, which was associated with cardiac hypertrophy, ventricular dilatation, increases in left ventricular mass and systolic and diastolic left ventricular internal diameters, and depressed contractile function [32]. Thirdly, immunoglobulin E (IgE) plays an important role in allergic asthma. Serum IgE level was highly increased in asthmatic patients compared to the control population [35]. High serum IgE level may activate IgE-FcεR1 pathway in cardiomyocytes and myocardial fibroblasts and plays a causative role in pathological cardiac remodeling and dysfunction by promoting cardiomyocyte hypertrophy, myocardial fibroblast activation, and matrix protein production [36]. The cardiac remodeling process is a common mechanism for the progression of heart failure [37]. Finally, obesity has been reported to play a role in both asthma and heart failure [38–40]. Evidence suggests that obesity is a state of low-grade inflammation, which releases cytokines that are associated with both asthma and cardiovascular diseases [41, 42]. In addition, the accumulation of abdominal and thoracic fat can have mechanical effects in chest wall expansion and lead to lung function impairment in asthma patients [43], which is also associated with higher risk of heart failure [44]. There is also accumulating evidence showing that anti-asthma medications including β2-agonists, anticholinergic agents, corticosteroids, leukotriene modifiers and others can affect the outcome of heart failure [45, 46]. For example, Salpeter and colleagues have shown that β-agonists use in patients with asthma increases the risk of congestive heart failure [45]. But another study indicated beneficial effect of inhaled β-adrenergic agonist albuterol in heart failure patients with preserved ejection fraction [46]. In summary, the overall effects of anti-asthma medications on heart failure depend on the stage of the disease, patient status, and routes of drug administration.

Previous research has suggested that atopic dermatitis is an allergic disease in which systemic inflammation involves more than just the skin [47]. A number of studies have examined a possible link between atopic dermatitis and various cardiovascular conditions [48, 49 Tamagawa-Mineoka, 2008 #114 Tamagawa-Mineoka, 2008 #114, 50]. So far there are various potential explanations for associations between atopic dermatitis and heart failure. First of all, systemic inflammation associated with atopic dermatitis may increase reactive oxygen species production in cardiac endothelial cells. The subsequent decrease in nitric oxide (NO) in dysfunctional endothelial cells resulted in low Protein kinsase G (PKG) activity in adjacent cardiomyocytes. Deficient NO-cGMP-PKG signaling from endothelium to myocardium not only promotes cardiomyocyte hypertrophy and interstitial fibrosis [48], but also affects myocardial relaxation and myocardial stiffness. High left ventricular myocardial diastolic stiffness is distinctively noticed in heart failure with preserved ejection fraction (HFpEF) [49]. Secondly, NO is a central regulator of platelet activation. Platelets activation has been demonstrated to be involved in the pathogenesis of atopic dermatitis. Plasma markers of platelet activation such as platelet-derived microparticles (PDMPs), soluble P-selectin (sP-selectin), and beta-thromboglobulin (beta-TG) were all remarkably increased in atopic dermatitis patients compared with healthy controls [50, 51]. Platelets activation may induce pathology in heart failure via C-C chemokines secretion [52]. Circulating levels of C-C chemokines have been proved to be increased in chronic heart failure and significantly correlated with the severity of symptoms and with the degree of left ventricular dysfunction [52]. Further studies are required to further delineate the direct pathogenic link between atopic dermatitis and heart failure.

We acknowledge the limitations of our study. First of all, although the sample size for heart failure GWAS is large, the statistical power can be further improved by reducing the heterogeneity of etiology and clinical manifestation of heart failure [16]. Secondly, lack of asthma subtypes for MR evaluation, since the current asthma subtype GWAS data with large-sample size are mainly coming from UK Biobank [12, 13, 53–56], which will lead to inflation of the MR results due to sample overlap between asthma subtypes or atopic dermatitis subtypes and heart failure. Thirdly, the current study restricted to the European population given the availability of GWAS data, thus the generalizability is limited. Future studies in non-European population are recommended to explore the causal association and heterogeneity in non-European population [57]. Fourthly, the current study used the binary variables as the exposure for the Mendelian randomization, which potentially has caveats [58]. The GWAS summary statistics for continuous variable, such as IgE level, is not publicly available. Future studies using continuous variable as the exposure is highly recommended. Fifthly, there are potential unmeasured confounders that may bias the Mendelian randomization results, such as obesity. We have evaluated the association of the SNPs in our instrumental variables with an independent body mass index GWAS [59], and did not observe the significant association (S6 and S7 Tables). Lastly, the current study restricted to evaluate the causal relationship of allergic diseases and heart failure based on genetic contribution, future studies will be needed to evaluate the environmental contribution (e.g., diet, life style) to both allergic diseases and heart failure.

In conclusion, our study identified asthma and atopic dermatitis as a causal risk factor for heart failure and suggest inflammatory pathogenesis as a key factor contributing to the underlying mechanism. These findings emphasize the importance of asthma and allergy control in the prevention and management of heart failure.

## Supporting information

S1 Table Summary of GWAS data. (DOCX)

S2 Table The characteristic of asthma associated index SNPs used as instrumental variable. (DOCX)

S3 Table The characteristic of atopic dermatitis associated index SNPs used as instrumental variable. (DOCX)

S4 Table Leave-one-out sensitivity analysis for Mendelian randomization analysis of asthma and heart failure. (DOCX)

S5 Table Leave-one-out sensitivity analysis for Mendelian randomization analysis of atopic dermatitis and heart failure. (DOCX)

S6 Table Association of asthma instrumental variable SNPs with body mass index GWAS. (DOCX)

S7 Table Association of atopic dermatitis instrumental variable SNPs with body mass index GWAS. (DOCX)
