# Supplementary material for: The causal relationship between allergic diseases and heart failure: Evidence from Mendelian randomization study
Source: PLoS One. 2022 Jul 29;17(7):e0271985. doi: 10.1371/journal.pone.0271985 (PMC9337678; doi:10.1371/journal.pone.0271985)
Supplement: S1 Table — (DOCX) [file pone.0271985.s001.docx]

Supplementary Table 1. Summary of GWAS data

| Phenotype | Data source | Case N | Control N | Total N | Genome build | Imputation panel | Population | # SNPs |
| --- | --- | --- | --- | --- | --- | --- | --- | --- |
| Asthma | Demenais et al., *Nature Genetics* 2018. | 19,954 | 107,715 | 127,669 | hg19 | HapMap 2 | European | 2,001,280 |
| Atopic dermatitis | Paternoster et al., *Nature Genetics* 2015. | 18,900 | 84,166 | 103,066 | hg19 | 1000G | European | 11,296,420 |
| Heart failure | Shah et al., *Nature Communications* 2020. | 47,309 | 930,014 | 977,323 | hg19 | Multiple | European | 8,281,262 |
