# Supplementary material for: The causal relationship between allergic diseases and heart failure: Evidence from Mendelian randomization study
Source: PLoS One. 2022 Jul 29;17(7):e0271985. doi: 10.1371/journal.pone.0271985 (PMC9337678; doi:10.1371/journal.pone.0271985)
Supplement: S2 Table — (DOCX) [file pone.0271985.s002.docx]

Supplementary Table 2. The characteristic of asthma associated index SNPs used as instrumental variable.

| SNP | Effect allele | Other allele | Effect allele frequency | Effect estimate | SE | P | N | Palindromic |
| --- | --- | --- | --- | --- | --- | --- | --- | --- |
| rs1032070 | T | C | 0.25 | -0.06 | 0.01 | 7.61E-06 | 127669 | No |
| rs10410595 | C | T | 0.14 | 0.08 | 0.02 | 8.48E-06 | 127669 | No |
| rs10455025 | C | A | 0.37 | 0.14 | 0.01 | 2.03E-25 | 127669 | No |
| rs10519067 | A | G | 0.14 | -0.12 | 0.02 | 1.49E-10 | 127669 | No |
| rs109156 | G | A | 0.45 | -0.06 | 0.01 | 5.91E-06 | 127669 | No |
| rs10957979 | G | A | 0.65 | -0.07 | 0.01 | 2.33E-08 | 127669 | No |
| rs10986311 | C | T | 0.34 | 0.06 | 0.01 | 4.52E-06 | 127669 | No |
| rs11583969 | T | C | 0.06 | 0.20 | 0.04 | 3.33E-06 | 127669 | No |
| rs11684791 | C | A | 0.38 | 0.06 | 0.01 | 4.53E-06 | 127669 | No |
| rs11686294 | A | G | 0.46 | 0.06 | 0.01 | 3.89E-06 | 127669 | No |
| rs11751184 | A | G | 0.27 | -0.07 | 0.02 | 6.91E-06 | 127669 | No |
| rs12245880 | T | C | 0.16 | 0.08 | 0.02 | 1.26E-06 | 127669 | No |
| rs12412656 | G | A | 0.28 | -0.07 | 0.01 | 1.53E-06 | 127669 | No |
| rs12728740 | A | C | 0.35 | -0.07 | 0.01 | 1.47E-07 | 127669 | No |
| rs12935657 | A | G | 0.25 | -0.10 | 0.01 | 2.06E-12 | 127669 | No |
| rs155585 | C | T | 0.92 | 0.12 | 0.02 | 7.34E-07 | 127669 | No |
| rs1663687 | A | G | 0.42 | -0.08 | 0.01 | 1.52E-10 | 127669 | No |
| rs167769 | T | C | 0.34 | 0.08 | 0.01 | 5.50E-09 | 127669 | No |
| rs16944061 | T | C | 0.02 | 0.18 | 0.04 | 2.13E-06 | 127669 | No |
| rs17293632 | T | C | 0.22 | 0.12 | 0.01 | 8.81E-16 | 127669 | No |
| rs174627 | A | G | 0.15 | -0.09 | 0.02 | 4.86E-07 | 127669 | No |
| rs20541 | G | A | 0.79 | -0.12 | 0.02 | 1.36E-14 | 127669 | No |
| rs2155219 | T | G | 0.49 | 0.11 | 0.01 | 2.90E-15 | 127669 | No |
| rs2305479 | T | C | 0.51 | -0.18 | 0.01 | 1.00E-42 | 127669 | No |
| rs2325291 | A | G | 0.36 | -0.10 | 0.01 | 8.58E-13 | 127669 | No |
| rs2327221 | A | G | 0.24 | 0.07 | 0.01 | 5.64E-06 | 127669 | No |
| rs2457382 | C | T | 0.66 | 0.07 | 0.01 | 3.04E-06 | 127669 | No |
| rs2646437 | A | G | 0.47 | -0.06 | 0.01 | 9.36E-06 | 127669 | No |
| rs2889896 | T | C | 0.49 | -0.06 | 0.01 | 1.03E-06 | 127669 | No |
| rs346835 | T | C | 0.33 | -0.07 | 0.01 | 1.77E-06 | 127669 | No |
| rs3751841 | T | G | 0.19 | -0.08 | 0.02 | 1.85E-06 | 127669 | No |
| rs3766568 | A | G | 0.33 | -0.06 | 0.01 | 5.38E-06 | 127669 | No |
| rs3771180 | T | G | 0.15 | -0.17 | 0.02 | 1.47E-20 | 127669 | No |
| rs3897686 | C | T | 0.55 | 0.06 | 0.01 | 8.38E-06 | 127669 | No |
| rs3936838 | C | T | 0.06 | 0.12 | 0.03 | 7.98E-06 | 127669 | No |
| rs4129267 | T | C | 0.35 | 0.06 | 0.01 | 6.29E-07 | 127669 | No |
| rs4447768 | T | C | 0.98 | -0.29 | 0.06 | 3.95E-06 | 127669 | No |
| rs4735849 | G | A | 0.19 | 0.08 | 0.02 | 5.14E-06 | 127669 | No |
| rs4742756 | A | G | 0.22 | 0.07 | 0.02 | 2.64E-06 | 127669 | No |
| rs500207 | G | A | 0.49 | -0.06 | 0.01 | 9.57E-06 | 127669 | No |
| rs6770872 | G | A | 0.54 | -0.06 | 0.01 | 2.67E-06 | 127669 | No |
| rs6851685 | T | G | 0.28 | -0.07 | 0.02 | 7.17E-06 | 127669 | No |
| rs6893213 | T | C | 0.10 | 0.15 | 0.02 | 7.99E-11 | 127669 | No |
| rs6906021 | C | T | 0.46 | 0.10 | 0.01 | 1.08E-13 | 127669 | No |
| rs6919792 | A | G | 0.69 | 0.06 | 0.01 | 9.44E-06 | 127669 | No |
| rs7209400 | T | C | 0.53 | 0.07 | 0.01 | 1.15E-08 | 127669 | No |
| rs7599342 | T | C | 0.49 | 0.07 | 0.01 | 7.79E-07 | 127669 | No |
| rs7694450 | G | A | 0.57 | 0.06 | 0.01 | 8.22E-06 | 127669 | No |
| rs7705042 | A | C | 0.62 | 0.08 | 0.01 | 8.53E-10 | 127669 | No |
| rs7961554 | C | A | 0.32 | 0.06 | 0.01 | 1.89E-06 | 127669 | No |
| rs841462 | C | T | 0.04 | 0.15 | 0.03 | 7.09E-06 | 127669 | No |
| rs881375 | C | T | 0.64 | -0.06 | 0.01 | 9.16E-06 | 127669 | No |
| rs9268969 | T | C | 0.32 | 0.14 | 0.01 | 5.29E-22 | 127669 | No |
| rs9546538 | C | T | 0.26 | 0.07 | 0.01 | 3.88E-06 | 127669 | No |
| rs992969 | G | A | 0.75 | -0.16 | 0.01 | 4.27E-29 | 127669 | No |
