# Supplementary material for: The causal relationship between allergic diseases and heart failure: Evidence from Mendelian randomization study
Source: PLoS One. 2022 Jul 29;17(7):e0271985. doi: 10.1371/journal.pone.0271985 (PMC9337678; doi:10.1371/journal.pone.0271985)
Supplement: S3 Table — (DOCX) [file pone.0271985.s003.docx]

Supplementary Table 3. The characteristic of atopic dermatitis associated index SNPs used as instrumental variable.

| SNP | Effect allele | Other allele | Effect allele frequency | Effect estimate | SE | P | N | Palindromic |
| --- | --- | --- | --- | --- | --- | --- | --- | --- |
| rs10200487 | T | C | 0.05 | 0.18 | 0.04 | 9.02E-06 | 33097 | No |
| rs1038165 | T | C | 0.57 | 0.08 | 0.02 | 3.04E-06 | 40530 | No |
| rs10790275 | C | G | 0.81 | 0.12 | 0.02 | 2.16E-08 | 40834 | No |
| rs111375762 | G | T | 0.04 | 0.26 | 0.05 | 7.58E-07 | 32814 | No |
| rs11156881 | T | C | 0.24 | -0.11 | 0.02 | 7.39E-07 | 34564 | No |
| rs11256611 | A | C | 0.13 | 0.11 | 0.02 | 4.34E-06 | 40833 | No |
| rs11581328 | A | G | 0.21 | -0.12 | 0.02 | 3.56E-07 | 34868 | No |
| rs11738721 | A | G | 0.42 | -0.10 | 0.02 | 1.02E-07 | 40529 | No |
| rs12144049 | T | C | 0.73 | -0.20 | 0.02 | 2.80E-27 | 40530 | No |
| rs12188917 | C | T | 0.21 | 0.17 | 0.02 | 2.89E-15 | 40530 | No |
| rs12334935 | A | G | 0.48 | 0.09 | 0.02 | 4.18E-08 | 40529 | No |
| rs12370257 | A | G | 0.08 | -0.15 | 0.03 | 8.28E-06 | 36934 | No |
| rs13152362 | A | G | 0.21 | -0.12 | 0.02 | 5.45E-08 | 40531 | No |
| rs13266315 | A | G | 0.60 | -0.08 | 0.02 | 1.09E-06 | 40834 | No |
| rs132911 | T | C | 0.74 | -0.09 | 0.02 | 6.62E-06 | 40833 | No |
| rs13302629 | A | G | 0.25 | -0.10 | 0.02 | 4.75E-06 | 37237 | No |
| rs13419662 | A | G | 0.30 | -0.09 | 0.02 | 4.53E-07 | 40529 | No |
| rs1347729 | A | C | 0.33 | -0.08 | 0.02 | 8.27E-06 | 40530 | No |
| rs144143913 | G | A | 0.02 | 0.45 | 0.09 | 1.18E-06 | 34256 | No |
| rs145009390 | G | C | 0.02 | 0.33 | 0.07 | 1.37E-06 | 34564 | No |
| rs145614235 | T | C | 0.03 | 0.27 | 0.06 | 6.96E-06 | 34867 | No |
| rs149553596 | G | T | 0.03 | 0.25 | 0.06 | 9.98E-06 | 34563 | No |
| rs181628386 | C | T | 0.01 | 0.48 | 0.10 | 2.42E-06 | 32242 | No |
| rs1857164 | G | A | 0.69 | 0.09 | 0.02 | 8.70E-06 | 40833 | No |
| rs188557945 | G | A | 0.98 | -0.63 | 0.14 | 4.29E-06 | 14400 | No |
| rs2041733 | C | T | 0.55 | -0.08 | 0.02 | 1.92E-06 | 40835 | No |
| rs2064330 | T | C | 0.06 | 0.22 | 0.05 | 6.20E-06 | 35182 | No |
| rs2155855 | G | A | 0.05 | 0.18 | 0.04 | 9.76E-06 | 37238 | No |
| rs2212434 | T | C | 0.45 | 0.13 | 0.02 | 2.09E-14 | 40530 | No |
| rs2433192 | A | G | 0.52 | -0.09 | 0.02 | 2.69E-07 | 40529 | No |
| rs2581790 | C | T | 0.32 | 0.09 | 0.02 | 1.42E-06 | 40530 | No |
| rs280729 | G | A | 0.18 | 0.11 | 0.02 | 1.38E-06 | 40530 | No |
| rs28507580 | G | A | 0.41 | 0.10 | 0.02 | 5.87E-06 | 29118 | No |
| rs2918299 | T | C | 0.17 | 0.14 | 0.02 | 5.45E-10 | 38477 | No |
| rs3120745 | G | A | 0.72 | -0.11 | 0.02 | 4.73E-08 | 40530 | No |
| rs3868879 | G | A | 0.70 | -0.08 | 0.02 | 6.42E-06 | 40529 | No |
| rs4151657 | C | T | 0.34 | 0.10 | 0.02 | 7.86E-09 | 40531 | No |
| rs479844 | G | A | 0.57 | 0.14 | 0.02 | 3.45E-17 | 40531 | No |
| rs4913279 | C | T | 0.63 | 0.08 | 0.02 | 2.94E-06 | 40529 | No |
| rs4976685 | G | A | 0.34 | 0.08 | 0.02 | 3.97E-06 | 40833 | No |
| rs530401 | G | A | 0.10 | 0.14 | 0.03 | 5.37E-06 | 34565 | No |
| rs6062486 | A | G | 0.69 | 0.10 | 0.02 | 2.40E-08 | 40531 | No |
| rs61850526 | T | C | 0.03 | 0.27 | 0.06 | 6.73E-07 | 34563 | No |
| rs6419573 | C | T | 0.76 | -0.12 | 0.02 | 2.92E-10 | 40529 | No |
| rs7226136 | C | A | 0.05 | 0.18 | 0.04 | 6.11E-06 | 40834 | No |
| rs7700687 | T | C | 0.61 | 0.09 | 0.02 | 3.74E-07 | 40529 | No |
| rs79030114 | T | A | 0.02 | 0.31 | 0.07 | 3.29E-06 | 34564 | No |
| rs7943728 | A | G | 0.16 | 0.11 | 0.03 | 7.57E-06 | 34563 | No |
| rs79739949 | T | C | 0.04 | 0.23 | 0.05 | 7.10E-06 | 34564 | No |
| rs8006 | C | G | 0.39 | 0.09 | 0.02 | 6.37E-07 | 40531 | No |
| rs8066625 | A | G | 0.11 | 0.18 | 0.03 | 3.84E-08 | 40529 | No |
| rs8090653 | C | T | 0.48 | -0.08 | 0.02 | 5.08E-06 | 36933 | No |
| rs906363 | C | T | 0.15 | 0.11 | 0.02 | 1.52E-06 | 40531 | No |
| rs9720781 | C | T | 0.58 | 0.09 | 0.02 | 9.39E-06 | 30427 | No |
