# Supplementary material for: The causal relationship between allergic diseases and heart failure: Evidence from Mendelian randomization study
Source: PLoS One. 2022 Jul 29;17(7):e0271985. doi: 10.1371/journal.pone.0271985 (PMC9337678; doi:10.1371/journal.pone.0271985)
Supplement: S4 Table — (DOCX) [file pone.0271985.s004.docx]

Supplementary Table 4. Leave-one-out sensitivity analysis for Mendelian randomization analysis of asthma and heart failure

| SNP | Effect estimate | SE | P |
| --- | --- | --- | --- |
| rs1032070 | 0.04 | 0.02 | 0.03 |
| rs10410595 | 0.04 | 0.02 | 0.03 |
| rs10455025 | 0.04 | 0.02 | 0.03 |
| rs10519067 | 0.04 | 0.02 | 0.02 |
| rs109156 | 0.04 | 0.02 | 0.03 |
| rs10957979 | 0.04 | 0.02 | 0.03 |
| rs10986311 | 0.04 | 0.02 | 0.03 |
| rs11583969 | 0.04 | 0.02 | 0.01 |
| rs11684791 | 0.04 | 0.02 | 0.02 |
| rs11686294 | 0.04 | 0.02 | 0.03 |
| rs11751184 | 0.04 | 0.02 | 0.03 |
| rs12245880 | 0.04 | 0.02 | 0.03 |
| rs12412656 | 0.04 | 0.02 | 0.01 |
| rs12728740 | 0.03 | 0.02 | 0.04 |
| rs12935657 | 0.04 | 0.02 | 0.02 |
| rs155585 | 0.04 | 0.02 | 0.03 |
| rs1663687 | 0.04 | 0.02 | 0.03 |
| rs167769 | 0.04 | 0.02 | 0.03 |
| rs16944061 | 0.04 | 0.02 | 0.03 |
| rs17293632 | 0.05 | 0.02 | 0.01 |
| rs174627 | 0.04 | 0.02 | 0.02 |
| rs20541 | 0.04 | 0.02 | 0.02 |
| rs2155219 | 0.04 | 0.02 | 0.04 |
| rs2305479 | 0.03 | 0.02 | 0.13 |
| rs2325291 | 0.04 | 0.02 | 0.04 |
| rs2327221 | 0.04 | 0.02 | 0.02 |
| rs2457382 | 0.04 | 0.02 | 0.02 |
| rs2646437 | 0.04 | 0.02 | 0.02 |
| rs2889896 | 0.04 | 0.02 | 0.03 |
| rs346835 | 0.04 | 0.02 | 0.03 |
| rs3751841 | 0.04 | 0.02 | 0.04 |
| rs3766568 | 0.04 | 0.02 | 0.02 |
| rs3771180 | 0.04 | 0.02 | 0.03 |
| rs3897686 | 0.04 | 0.02 | 0.02 |
| rs3936838 | 0.04 | 0.02 | 0.03 |
| rs4129267 | 0.04 | 0.02 | 0.02 |
| rs4447768 | 0.04 | 0.02 | 0.02 |
| rs4735849 | 0.04 | 0.02 | 0.04 |
| rs4742756 | 0.04 | 0.02 | 0.03 |
| rs500207 | 0.04 | 0.02 | 0.03 |
| rs6770872 | 0.04 | 0.02 | 0.03 |
| rs6851685 | 0.04 | 0.02 | 0.02 |
| rs6893213 | 0.04 | 0.02 | 0.03 |
| rs6906021 | 0.04 | 0.02 | 0.02 |
| rs6919792 | 0.04 | 0.02 | 0.03 |
| rs7209400 | 0.04 | 0.02 | 0.03 |
| rs7599342 | 0.04 | 0.02 | 0.02 |
| rs7694450 | 0.04 | 0.02 | 0.03 |
| rs7705042 | 0.04 | 0.02 | 0.04 |
| rs7961554 | 0.04 | 0.02 | 0.01 |
| rs841462 | 0.04 | 0.02 | 0.03 |
| rs881375 | 0.04 | 0.02 | 0.03 |
| rs9268969 | 0.04 | 0.02 | 0.03 |
| rs9546538 | 0.04 | 0.02 | 0.03 |
| rs992969 | 0.04 | 0.02 | 0.04 |
| All | 0.04 | 0.02 | 0.03 |
