# Supplementary material for: The causal relationship between allergic diseases and heart failure: Evidence from Mendelian randomization study
Source: PLoS One. 2022 Jul 29;17(7):e0271985. doi: 10.1371/journal.pone.0271985 (PMC9337678; doi:10.1371/journal.pone.0271985)
Supplement: S5 Table — (DOCX) [file pone.0271985.s005.docx]

Supplementary Table 5. Leave-one-out sensitivity analysis for Mendelian randomization analysis of atopic dermatitis and heart failure

| SNP | Effect estimate | SE | P |
| --- | --- | --- | --- |
| rs10200487 | 0.03 | 0.01 | 0.02 |
| rs1038165 | 0.03 | 0.01 | 0.01 |
| rs10790275 | 0.04 | 0.01 | 0.01 |
| rs111375762 | 0.03 | 0.01 | 0.01 |
| rs11156881 | 0.03 | 0.01 | 0.01 |
| rs11256611 | 0.03 | 0.01 | 0.02 |
| rs11581328 | 0.03 | 0.01 | 0.02 |
| rs11738721 | 0.03 | 0.01 | 0.01 |
| rs12144049 | 0.03 | 0.01 | 0.01 |
| rs12188917 | 0.03 | 0.01 | 0.01 |
| rs12334935 | 0.04 | 0.01 | 0.00 |
| rs12370257 | 0.03 | 0.01 | 0.01 |
| rs13152362 | 0.03 | 0.01 | 0.01 |
| rs13266315 | 0.03 | 0.01 | 0.02 |
| rs132911 | 0.03 | 0.01 | 0.02 |
| rs13302629 | 0.03 | 0.01 | 0.01 |
| rs13419662 | 0.03 | 0.01 | 0.01 |
| rs1347729 | 0.03 | 0.01 | 0.02 |
| rs144143913 | 0.03 | 0.01 | 0.01 |
| rs145009390 | 0.03 | 0.01 | 0.01 |
| rs145614235 | 0.04 | 0.01 | 0.00 |
| rs149553596 | 0.03 | 0.01 | 0.02 |
| rs181628386 | 0.03 | 0.01 | 0.02 |
| rs1857164 | 0.03 | 0.01 | 0.02 |
| rs188557945 | 0.04 | 0.01 | 0.00 |
| rs2041733 | 0.03 | 0.01 | 0.01 |
| rs2064330 | 0.03 | 0.01 | 0.01 |
| rs2155855 | 0.03 | 0.01 | 0.01 |
| rs2212434 | 0.03 | 0.01 | 0.02 |
| rs2433192 | 0.03 | 0.01 | 0.02 |
| rs2581790 | 0.03 | 0.01 | 0.02 |
| rs280729 | 0.03 | 0.01 | 0.02 |
| rs28507580 | 0.03 | 0.01 | 0.01 |
| rs2918299 | 0.03 | 0.01 | 0.02 |
| rs3120745 | 0.04 | 0.01 | 0.01 |
| rs3868879 | 0.03 | 0.01 | 0.01 |
| rs4151657 | 0.03 | 0.01 | 0.02 |
| rs479844 | 0.03 | 0.01 | 0.03 |
| rs4913279 | 0.03 | 0.01 | 0.02 |
| rs4976685 | 0.03 | 0.01 | 0.01 |
| rs530401 | 0.03 | 0.01 | 0.02 |
| rs6062486 | 0.03 | 0.01 | 0.01 |
| rs61850526 | 0.03 | 0.01 | 0.02 |
| rs6419573 | 0.03 | 0.01 | 0.01 |
| rs7226136 | 0.03 | 0.01 | 0.01 |
| rs7700687 | 0.03 | 0.01 | 0.02 |
| rs79030114 | 0.03 | 0.01 | 0.01 |
| rs7943728 | 0.04 | 0.01 | 0.01 |
| rs79739949 | 0.03 | 0.01 | 0.01 |
| rs8006 | 0.03 | 0.01 | 0.01 |
| rs8066625 | 0.03 | 0.01 | 0.02 |
| rs8090653 | 0.03 | 0.01 | 0.01 |
| rs906363 | 0.03 | 0.01 | 0.01 |
| rs9720781 | 0.03 | 0.01 | 0.01 |
| All | 0.03 | 0.01 | 0.01 |
