# Supplementary material for: The causal relationship between allergic diseases and heart failure: Evidence from Mendelian randomization study
Source: PLoS One. 2022 Jul 29;17(7):e0271985. doi: 10.1371/journal.pone.0271985 (PMC9337678; doi:10.1371/journal.pone.0271985)
Supplement: S6 Table — (DOCX) [file pone.0271985.s006.docx]

Supplementary Table 6. Association of asthma instrumental variable SNPs with body mass index GWAS

| SNP | P-value | N |
| --- | --- | --- |
| rs10986311 | 0.001901 | 233739 |
| rs12245880 | 0.002091 | 233643 |
| rs3751841 | 0.00327 | 221908 |
| rs2889896 | 0.008751 | 233864 |
| rs167769 | 0.03227 | 321771 |
| rs4129267 | 0.03269 | 322092 |
| rs7694450 | 0.09393 | 232977 |
| rs2305479 | 0.1563 | 321937 |
| rs4742756 | 0.1576 | 233957 |
| rs6851685 | 0.1649 | 318099 |
| rs109156 | 0.1691 | 221916 |
| rs881375 | 0.1972 | 233951 |
| rs9546538 | 0.2009 | 233996 |
| rs1032070 | 0.207 | 233872 |
| rs20541 | 0.2094 | 222168 |
| rs3771180 | 0.2147 | 230332 |
| rs2327221 | 0.2464 | 233912 |
| rs6906021 | 0.2583 | 313457 |
| rs174627 | 0.2617 | 321947 |
| rs10455025 | 0.2713 | 233575 |
| rs12728740 | 0.2824 | 233750 |
| rs7961554 | 0.2895 | 322033 |
| rs9268969 | 0.3293 | 232596 |
| rs7599342 | 0.3296 | 232865 |
| rs11751184 | 0.3514 | 233735 |
| rs6770872 | 0.3709 | 222258 |
| rs4735849 | 0.3752 | 228724 |
| rs3766568 | 0.4237 | 233947 |
| rs7209400 | 0.4332 | 233834 |
| rs500207 | 0.4612 | 229854 |
| rs4447768 | 0.4793 | 180641 |
| rs2646437 | 0.4938 | 232639 |
| rs1663687 | 0.4997 | 233997 |
| rs7705042 | 0.5106 | 234018 |
| rs16944061 | 0.5285 | 211605 |
| rs6893213 | 0.5524 | 230682 |
| rs11583969 | 0.5864 | 288331 |
| rs346835 | 0.6085 | 232279 |
| rs6919792 | 0.6089 | 233618 |
| rs10519067 | 0.6171 | 225824 |
| rs841462 | 0.6251 | 230082 |
| rs2325291 | 0.6348 | 233863 |
| rs10410595 | 0.6507 | 229721 |
| rs2457382 | 0.7077 | 233716 |
| rs3936838 | 0.7236 | 233727 |
| rs992969 | 0.7272 | 233958 |
| rs12412656 | 0.7328 | 231841 |
| rs17293632 | 0.8342 | 232007 |
| rs155585 | 0.8415 | 233909 |
| rs11684791 | 0.8644 | 206478 |
| rs2155219 | 0.8745 | 233482 |
| rs3897686 | 0.9162 | 233646 |
| rs11686294 | 0.9569 | 233668 |
| rs10957979 | 0.979 | 233815 |
| rs12935657 | 0.9814 | 233346 |

N: sample size of body mass index GWAS
