# Supplementary material for: The causal relationship between allergic diseases and heart failure: Evidence from Mendelian randomization study
Source: PLoS One. 2022 Jul 29;17(7):e0271985. doi: 10.1371/journal.pone.0271985 (PMC9337678; doi:10.1371/journal.pone.0271985)
Supplement: S7 Table — (DOCX) [file pone.0271985.s007.docx]

Supplementary Table 7. Association of atopic dermatitis instrumental variable SNPs with body mass index GWAS

| SNP | P-value | N |
| --- | --- | --- |
| rs8006 | 0.001004 | 234014 |
| rs2155855 | 0.1687 | 233827 |
| rs4913279 | 0.1852 | 231892 |
| rs11581328 | 0.2094 | 233774 |
| rs4151657 | 0.2354 | 320667 |
| rs2918299 | 0.2656 | 222075 |
| rs13152362 | 0.2686 | 233781 |
| rs2581790 | 0.3421 | 233819 |
| rs906363 | 0.3642 | 220397 |
| rs9720781 | 0.3754 | 170379 |
| rs3868879 | 0.407 | 233753 |
| rs10200487 | 0.455 | 57095 |
| rs2041733 | 0.4902 | 322076 |
| rs4976685 | 0.5157 | 233791 |
| rs1347729 | 0.6348 | 233966 |
| rs12144049 | 0.6418 | 233769 |
| rs7226136 | 0.8026 | 233575 |
| rs13266315 | 0.8128 | 233900 |
| rs479844 | 0.8288 | 233971 |
| rs13302629 | 0.8864 | 210546 |
| rs1038165 | NA | NA |
| rs10790275 | NA | NA |
| rs111375762 | NA | NA |
| rs11156881 | NA | NA |
| rs11256611 | NA | NA |
| rs11738721 | NA | NA |
| rs12188917 | NA | NA |
| rs12334935 | NA | NA |
| rs12370257 | NA | NA |
| rs132911 | NA | NA |
| rs13419662 | NA | NA |
| rs144143913 | NA | NA |
| rs145009390 | NA | NA |
| rs145614235 | NA | NA |
| rs149553596 | NA | NA |
| rs181628386 | NA | NA |
| rs1857164 | NA | NA |
| rs188557945 | NA | NA |
| rs2064330 | NA | NA |
| rs2212434 | NA | NA |
| rs2433192 | NA | NA |
| rs280729 | NA | NA |
| rs28507580 | NA | NA |
| rs3120745 | NA | NA |
| rs530401 | NA | NA |
| rs6062486 | NA | NA |
| rs61850526 | NA | NA |
| rs6419573 | NA | NA |
| rs7700687 | NA | NA |
| rs79030114 | NA | NA |
| rs7943728 | NA | NA |
| rs79739949 | NA | NA |
| rs8066625 | NA | NA |
| rs8090653 | NA | NA |

N: sample size of body mass index GWAS
